# Currencies of greater interest for central Asian economies: an analysis of exchange market pressure amid global and regional interdependence

**DOI:** 10.1186/s40854-022-00417-7

**Published:** 2023-01-19

**Authors:** Devendra Kumar Jain, Naqeeb Ur-Rehman, Omonjon Ganiev, Kapil Arora

**Affiliations:** 1 Flame School of Business, Flame University, Pune, India; 2 School of Economics, Westminster International University, Tashkent, Uzbekistan; 3 School of Business & Economics, Westminster International University in Tashkent, Tashkent, Uzbekistan; 4 Alliance School of Business, Alliance University, Bangalore, India

**Keywords:** Exchange market pressures, Central Asian economies, Exchange markets, Financial market Interdependence, Transition economies, G15, G18, G01, F42, E52

## Abstract

Central Asian Economies (CAEs) have diverse exchange rate policies. They have recorded higher volatility in the foreign exchange market since inception. High volatility of the transition era has drifted these economies towards partial dollarization. Monetary authorities in CAEs, (already have a challenge of maintaining monetary policy autonomy) have a gigantic task of price stability and stopping the spread of dollarization. This study is directed towards assessing the drivers and the determinants of foreign exchange market pressure in CAEs. The results, based on panel data analysis and the System GMM model, have provided useful insights about the exchange market pressure determinants particularly USD, Euro, Ruble, and Renminbi. The results show that China and Russia exchange market pressure has a negative effect on the exchange market pressure of CAEs. While the dollar index shows a positive impact on the exchange market pressure of CAEs. Overall, the findings imply that China and Russia currency appreciation results in a trade deficit across CAEs. The policy implication suggests that the floating exchange rate regime (inflation targeting regime) is not in favor of CAEs, and they must use managed-float to reduce their trade deficits.

## Introduction

Central Asia^1^ has many monetary policy challenges. The changing dynamics of world trade has increased the influence of large economies over smaller and transition economies. The monetary authorities of the transition economies of Central Asia have limited options to carry forward their monetary policy objectives. There has been high volatility in foreign exchange markets of Central Asian Economies (CAEs) since 2014. There was a policy discourse for CAE and the Caucasus to adapt greater exchange rate flexibility (Horton et al. 2016). But flexible exchange rate policy does not necessarily translate into the independence of monetary policy autonomy (Rey 2018). Financial shocks in the form of exchange rate pass-through have impacted transition economies and emerging economies across the globe irrespective of exchange rate regime, state of economy and trade links (Corsetti et al. 2021).

Transition economies (CAEs) have to devise an appropriate exchange rate policy or monetary policy that can address the issue of exchange rate pass-through, the changing dynamics of world trade with historically linked large economies and the development agenda. Moreover, due to a lack of credible exchange rate policy, CAEs have suffered a high rate of dollarization in the recent past. The de-dollarization is a high priority agenda of monetary policy reforms (Naceur et al. 2015). The export-oriented economies (trade surplus economies) can manage exchange rates to their advantage and a majority of these countries do not practice floating exchange rate regimes. The commodity currencies usually co-move with commodity prices and are less affected by financial shocks and contagion. The economies of reserve currencies have the advantage of issuing (printing) currencies to run huge deficits. None of these options is available to transition economies of the CAE.

This paper is set out in this background. What are monetary policy options for CAEs to formulate their exchange rate policies? Before knowing the answer to this question, It would be appropriate to develop our understanding of the interdependence of the CAE foreign exchange market with major trade and financial partners such as the US, Europe, Russia and China. In other words, to what extent, the exchange market pressures from these four countries (US, Europe, Russia and China) have amplified the foreign exchange markets in CAEs. Without solving this puzzle, we cannot have an optimal exchange rate policy and related macroeconomic stability.

The concept of optimum currency area to exchange rate regimes were part of the earlier discussion on the subject (Willett 2004). Small and medium-sized countries have to deal with the consequences of having a large currency area in a close neighbourhood to maintain monetary independence. The concept of financial market interdependence was critically discussed in earlier studies prominently in Li et al. (2012). Poghosyan (2021) has estimated exchange rate pass-through for the Caucasus and Central Asia Countries. To the best of our knowledge, there is a clear gap in the literature on the topic of the interdependence of foreign exchange markets of CAEs with four major trading blocks (US, Europe, Russia and China).

Our paper is predicated on the interdependence of foreign currency markets and the spillover effects of exchange rate movements of large trading economies on the foreign exchange markets and exchange rate policies of smaller economies with close trade and financial ties. The primary source of inspiration for this paper is the literature on the financial crisis of the 1990s. Recent research (Kim et al. 2015) has reaffirmed the impact of the US financial crisis on the financial asset return and foreign exchange market of Asian nations.

Our strategy stemmed from the earlier works that have used the Exchange market pressure index to measure financial crises such as (Li et al. 2006; Patnaik et al. 2017; Patnaik and Pundit 2019). The independent variables used in this study for interdependence have also found a place in earlier works such as (Berry et al. 2007; Ahmed et al. 2017).

Our unique contribution to the literature is the estimation of the Exchange Market Pressure Index for four Central Asian Economies.^2^ The issue of interdependence in the foreign exchange market of CAE with major trade blocks has been dealt with in detail for the first time in the literature. We have also discussed the growing pattern of trade among CAEs and with the big four for an intuitive understanding of the topic.

The remainder of the paper is organized as follows. Section 2 surveys the existing literature on the subject and clearly identifies the gap (lack of research on central Asian economies on the subject) though the existing literature widely supports our methodology and financial market linkages. In Sect. 3, we discuss the historical evolution of exchange rate policies of CAEs post transition era and the context of writing this paper. Section 4 explains the literature and methodology of the exchange market pressure index, a dependent variable in our model. The model and selection of variables are discussed in Sect. 5. We have chosen system GMM model as it provides efficient estimates for small sample size panel data, removes endogeneity, and tackles the causality issue in the model. In Sect. 6, we present the results and the analysis the robustness of results as Ruble and Renminbi coefficient are negative and significant. The widening trade gap of CAEs with Russia and China explains the robustness of the results. In concluding Sect. 7, we have discussed the policy implications of these results in context of monetary policy, international coordination & transmission, and needed government policy and regulations. As CAEs are moving towards inflation targeting monetary policy and liberalization of foreign exchange market, our results provide useful insights of interdependence with neighboring countries and major trading partners.

## Literature review

Volatility spillover, contagion effects of currency crises, co-movements in foreign exchange markets, exchange rate pass-through, and interdependence are related topics to our research question. Most economic variables don't have normal distributions and don't have linear relationships. This is why fat tails tend to happen in times of stress. Dynamics with cross variables enables for interactions. Given the econometric link, the shock to one variable will then affect other variables. Macroeconomic similarities and financial market integration including the behaviour of global institutional investors are linked to contagion and spillover. The fundamental premise of our model is based on the fact that the exchange market pressures of Central Asian Economies are largely influenced by three types of factors such as global, regional and local. In accordance with our hypothesis, the dollar index represents a global factor, regional factors comprise EMP of China and EMP of Russia, and local variables include forex reserve, exchange rates, trade balance, and broad money. These factors are sometimes beyond the trade-weights. There are prior studies on the use of EMP to study the transmission of crisis from Thailand to neighbouring countries (Horen et al. 2006).

Initial studies in the literature have found trade and financial linkages in cross-markets. Eichengreen et al. (1995) argue that the initial propagation of financial shocks originates from trade channels. Glick and Rose (1999) have also confirmed that trade plays an important role in transmitting financial shocks to other countries. Forbes (2001) finds trade as the most important link in the transmission process. The financial linkages such as a common creditor, interconnected lender, interaction of market-driven financial system and portfolio rebalancing were found to be relevant while studying the causes of contagion. Forbes and Rigobon (1999) clarify that the contagion arises due to a shift in cross-market linkages which the authors define as ‘shift-contagion’. The authors further argue that if two markets have high comovements after a financial shock, that cannot be termed as contagion. It can be termed as shift-contagion only. There is a detailed explanation of the mechanism of transmitting volatility in the work of Dornbusch et al. (2000). These authors have opined that there is a need to look at the microeconomic considerations (such as capital flows, spillover of the volatility of exchange market, and common creditor) and institutional factors (role of banks and financial institutions, role of international financial agents, collective irrational behaviour of investors) in understanding the contagion and financial market volatility.

Gelos and Sahay (2001) have examined the issue of financial market spillovers from cross country data. Their findings suggest that indirect trade linkages appear to be more important than bilateral trade links. This study employs the exchange market pressure index of transition economies to test pairwise correlations and granger casualty. Dungey and Martin (2004) apply a multifactor model to estimate the impact of contagion on the volatilities of exchange rates during the East Asian currency crisis. Horen et al. (2006) examine the contagion of foreign exchange markets from Thailand to other neighbouring countries (Indonesia, Korea, Malaysia and the Philippines) using Exchange Market Pressure Index.

Gardini and Angelis (2012) suggest time-varying dynamics of conditional correlation (DCC) to test both endogenous and exogenous amplification processes of volatility in financial markets. They further measure equilibrium risk premia and the distance between equilibrium level and the actual risk premia. They have used a behavioural model for estimating equilibrium risk premium. Bua and Trecroci (2019) suggest that international equity market volatility changes with the perception of macroeconomic risk. They further highlight the fact that the high volatility spell of all indices coincides with macroeconomic slowdowns because expected cash flows (market valuations) are adjusted on the lines of expected changes in macroeconomic variables such as GDP growth, industrial production, policy interest rates and fiscal imbalances. Rigobon (2016) has compiled all the issues (three biases- endogeneity, omitted variables and heteroskedasticity) related to models of contagion and interdependence such as correlations, principal components, Ordinary Least Square (OLS) regressions Event studies, Probit -Logit and ARCH –GARCH. The author has presented theoretical aspects of contagion and spillover as fundamental views, financial views and coordination views. The concept of the bilateral currency crisis and its welfare cost in form of reduced bilateral trade has been innovatively discussed by Yilmazkuday (2021).

There are several studies on the interdependence of foreign exchange markets and foreign exchange rates for several currency pairs. While Booth et al. (2005) have primarily focused on long-term interdependence of 91 currency pairs, Yang et al. (2016) has used wavelet coherence analysis to differentiate short-term and long-term interdependence on returns of four major exchange rates. To capture the dependence structure of each currency pair, Richard (2020) has used a mixed copula approach.

Mody and Taylor (2007) have explored the regional component of exchange market pressure fore East Asian economies. The finding suggest that the trade links have little role in the regional exchange market pressure and external liabilities are more relevant to the exchange rate volatility. Aizenman and Hutchison (2012) have studied transmission mechanism of financial crises from US to emerging markets. They have analyzed two important components of exchange market pressure (EMP) changes in exchange rates and changes in international reserves. The emerging economies have given higher weight for changes in exchange rates (towards depreciation) than absorbing shocks by losing international reserves. Ahmed (2021) has raised the issue of classification of exchange rate regimes. While studying the trends in exchange rate policy of 52 countries, the author has emphasized the need for constant evaluation of exchange rate flexibility for estimating monetary policy spillover. There is a plethora of literature on increasing role of RMB (Yuan–Chinese currency) in the Asian financial markets due to increasing financial linkages apart from trade links (Arslanalp et al. 2016; Xiong and Han 2015; Shu et al. 2015).

According to a recent study by Wei et al. (2020), RMB fluctuations (China as a major regional player) have impacted financial reforms in B &R countries (“the Bell & Road” member countries). It has also served as an external shock to their monetary policies, such as volatility in the currency market. In our paper, we attempt to expand on this analysis by incorporating all currencies of CAEs' major trading partners and developing an analytical tool to address foreign exchange market pressures and appropriate exchange rate policies in CAEs.

The sustainability of the US dollar as the dominant currency of international trade and financial transactions is a related area of research in the field of open economy macroeconomics. There is no real contender to replace the US dollar as the world's hegemonic monetary currency in the near future (Fields and Vernengo 2013). Since 2010, China has been promoting the Yuan as an international trade currency. This has had limited success. The ongoing Russia-Ukraine conflict has reignited debate over alternatives to the US dollar as an invoice currency for international trade. In addition, the Reserve Bank of India has opened a Rupee settlement window for international transactions. Gopinath et al. (2020) discussed the dominant currency paradigm (DCP) for international trade and reaffirmed the US dollar's dominance in global trade. Gevorkyan and Khemraj (2022) investigated the US dollar's dominance through exchange market pressure. According to the study, exchange market pressure is equivalent to foreign exchange liquidity. Because the US dollar is the dominant currency, it will have a clear impact on the real variables of producing and destination countries invoicing in dollars. Recent crashes in financial markets in emerging and developing economies, including crypto currencies, reaffirm the US dollar's role as the world's dominant currency. The US dollar has risen against all major world currencies. Interestingly, the Ruble has appreciated against major currencies to the prewar level defying the global trend. The trade surplus of Russia has reached to a record level in Q2,2022. The Q2 (2022) trade surplus figure has recorded $ 70 billion (the corresponding period figure was $ 17.3 billion only).

The approach in our research is somewhat different from earlier studies. Our context is to identify the stage of interdependence in CAEs with four major blocks (due to past and present linkages) for implementing an appropriate exchange rate policy in CAEs. In the following section, we review the historical development in exchange rates and related policies in CAEs.

## Foreign exchange market and policies in central Asian economies

Soon after the transition from the Soviet Union, CAEs have witnessed high inflation and increasing dollarization. CAEs have realized the pitfalls of dollarization and have implemented many reforms in the monetary policy framework. We have presented a sketch view of macroeconomic indicators of each CAE (“Appendix Table 4”). In conjecture with macroeconomic fundamentals, central banks across CAEs have taken an inflation-targeting approach to have price stability as a primary objective of their monetary policies. According to IMF (Areaer 2019), CAEs have declared (De Jure) their exchange rate regime as floating. But De facto, they are practicing either stabilized arrangement or crawl-like arrangements. Kazakhstan is most developed among CAEx with GNI Per capita is $8820 (WDI 2019). The other three countries have a range of GNI per capita $ 1000–1800. The National Bank of Kazakhstan (NBK) has implemented a new monetary policy with an inflation target of 5.7. The level of inflation has reached 7.4% towards the end of 2020 due to Covid-19. Uzbekistan is the second-largest country among CAEs has implemented all-around reforms in real sectors, liberalization of trade and reforms in foreign exchange markets by introducing inflation targeting regime. The short-term target for inflation is below 10% by end of 2021 and gradually moving toward a long-term target of 5% by 2023. The monetary authority of Kyrgyzstan has implemented an inflation targeting regime from 2018 with a short-term target of 5.7%. The post-pandemic period has witnessed a surge in inflation above 10%. A similar story can be seen in Tajikistan. The National Bank of Tajikistan has set an inflation target of 7.2% in the short-term (2021) and a long-term target of 6.2%. Inflation reached to 9.4% level after the pandemic (2020). The Central Bank of Uzbekistan (CBU) started inflation targeting in 2019 and set an immediate target at below 10% (by 2021) and a long-term target of 5% by 2023. Well before the pandemic, Uzbekistan has recorded inflation of 15% in 2019. Due to the pandemic, the CBU continued soft monetary policies and reduced the policy rate.^3^

**Fig. 1:**
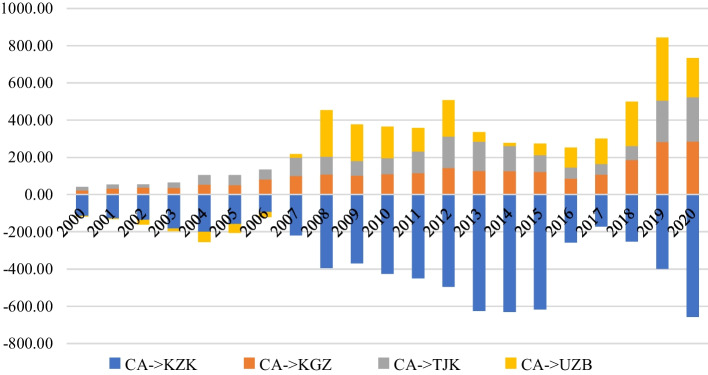
Intra-regional trade of Central Asia (million dollars)

A sketch view of All the inflation targeting policies of CAEs have failed to reduce or stabilize high volatility in their foreign exchange markets. This is one of the motives of this study from the policy perspective. We have investigated the trade links among CAEs (Fig. 1) to get the present state of the intra-trade among CAEs.

Figure 1 shows the intra-region trade across 4 central Asian countries as Kazakhstan, Kyrgyzstan, Tajikistan, and Uzbekistan. In Fig. 1, Kazakhstan shows a trade surplus against Kyrgyzstan, Tajikistan, and Uzbekistan. In other words, these three countries present a trade deficit against Kazakhstan. We can postulate that Kazakhstan is dominant in terms of trading with the remaining central Asian neighbouring countries. Similarly, Kyrgyzstan consistently shows a trade deficit while trading with the other three members. This trade deficit becomes large from 2018 to 2020. In sum, we can conclude that Kazakhstan has a relative trade advantage as compared to the other three members and it is because of the high natural resources of Kazakhstan.

**Fig. 2:**
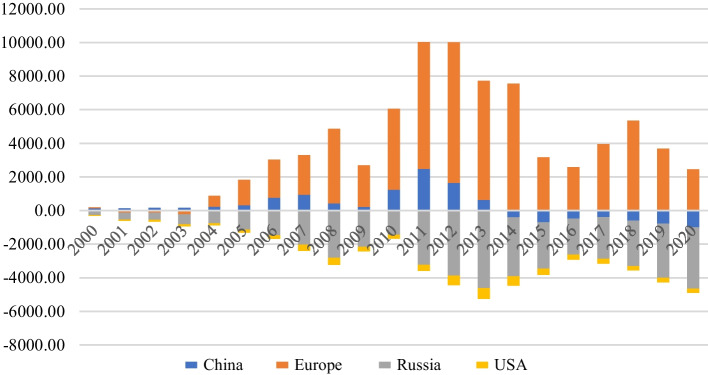
Central Asia direction of trade with others (million dollars)

To get intuitive knowledge of the interdependence of Foreign exchange markets of CAE with the big four (US, Europe, Russia and China), we have reviewed the trade balance of CAEs with the big four (Fig. 2). All four countries showed trade deficits against China and Russia and this trade deficit is widening.

## Methodology (theory, Variables, data sources and the empirical model)

### Theoretical description of exchange market pressure index (EMP)

The co-movements in asset prices finally reflect in the nominal exchange rate or international reserves of a country or policy interest rate individually or collectively. Therefore, a composite variable of these three i.e. EMP is a variable of much macroeconomic significance. The concept of EMP was first introduced by Girton and Roper (1977) to study the balance of payment crisis. The EMP, as a composite variable of changes in the exchange rate and changes in foreign exchange reserve, has extensively been used in financial and macroeconomic studies. The past studies focused on the balance of payment crisis, financial crises, and misalignment of the exchange rate, calculation of crisis index, calculation of intervention index, de facto exchange rate regimes and dynamics of exchange rate management. Few studies have also used econometric models to study the interaction of EMP with its determinants and other macroeconomic variables of an economy such as interest rate changes, net exports, credit growth and capital control. There are cross country studies as well as country-specific (Jain et al. 2022). Exchange Market Pressure as a dependent variable has been used in prominent studies such as Gelos and Sahay (2001), Pentecost et al. (2001), Horen et al. (2006), van Poeck et al. (2007), Patnaik et al. (2017), Ahmed et al. (2017) and Patnaik and Pundit (2019).

We have used the concept of EMP in a different dimension. Unlike many studies (quite a number), we do not consider fluctuations in exchange rates as an absolute sign of the volatility of distress in the foreign exchange market. Monetary authorities can use multiple monetary policy tools to manage the volatility of the exchange market. The more appropriate method of measuring distress in the foreign exchange market is the EMP that can measure pressure in any exchange rate regime or any size of the economy in a non-parametric way. Therefore, we have used the EMP to measure the interdependence or international spillover of foreign exchange market volatility.

As there are different objectives for estimating the exchange market pressure index, we have found many variations in the estimating models. Here, we are discussing three important models popularly used in the literature. The most popular model is called ERW 1997 model (Eichengreen et al. 1997). According to this model, the construction of the exchange market pressure index is to be done by a weighted average of all three variables (changes in the exchange rate, changes in international reserve (domestic and foreign) and changes in interest rate (domestic and foreign). The weighing scheme adopted here is to avoid the dominance of one variable against others as all three variables are different. This scheme equalizes the volatilities of the variables of EMP. The model-independent formula for calculation of EMP (called as ERW model)is as under:

1 $$EMP_{t } = \left[ { \left( { \alpha \% {\Delta } e_{t} } \right) + \left( { \beta {\Delta } \left( { i_{t} - i*_{t} } \right)} \right) - \left( { \gamma \left( {\% {\Delta }r _{t} - \% {\Delta }r* _{ t } } \right)} \right)} \right]$$

In this equation, e, is the exchange rate, i, is the interest rate and r, is the reserve. The interest rate in anchor country/foreign currency is i* and r* is to represent the reserve of anchor country/foreign currency. The weights (α, β, γ) are used to equalize the volatility of each component. Accordingly, weights are calculated as under:

2 $$\alpha = \frac{1}{{\sigma_{e } }}: \beta = \frac{1}{{\sigma_{r} }} : \gamma = \frac{1}{{\sigma_{i } }}$$

Here σe is the standard deviation (SD) of the exchange rate (ER), σ r (SD of reserves) and σ i (SD of Interest rate).

Sachs et al. (1996) modified the weighing scheme and introduced the concept of the inverse of the variance. The inverse method is calculated as follows:

3 $$\alpha = \frac{{1/\sigma_{e} }}{{\frac{1}{{\sigma_{e} }} + \frac{1}{{\sigma_{r} }} + \frac{1}{{\sigma_{i} }}}}$$

The inverse of variance for β and γ can be calculated analogously. The issue of equal weight and weighing through the inverse of variance have been contested by subsequent researchers for example, (Pontines and Siregar 2008; Klaassen 2012).

The concept of exchange market pressure index (EMPI) does not analyze changes in exchange rate as an isolated analysis of currency appreciation or depreciation. The design of EMPI consist of changes in exchange rate, changes in international reserves and changes in policy interest rates. The higher reading of the EMPI indicates a pressure for depreciation of the domestic currency and lower reading of index entails a pressure for appreciation of the domestic currency. Therefore EMPI considers exchange market pressure when there is wide fluctuations on either side. The crisis index is created from the EMPI to predict or forecast period of financial crises or balance of payment distresses (Patnaik and Pundit 2019; Aizenman and Binici 2016).

### Methodology used for exchange market pressure index of CAEs

As market-determined interest rates are not easily available for CAEs due to underdeveloped bond markets, the interest rate variable has been dropped in our estimation of the EMP. This strategy has been recommended by earlier researchers as well such as Sachs et al. (1996), Kaminsky et al. (1998) and Cardarelli et al. (2009). We have used Cardarelli et al. (2009) methodology with minor modifications. The EMP index is calculated as follows:

4 $$EMP_{t} = \frac{1}{{\sigma \Delta e_{t } }}\Delta e_{t } + \frac{1}{{\sigma \Delta r_{t} }}\Delta r_{t}$$

σ∆e (standard deviation of changes in the exchange rate) σ∆r (standard deviation of changes in reserves). One of the criticisms of the EMP methodology is about static elements in the weight calculations. We introduced a time-varying concept in the estimation of the EMP. Instead of taking one standard deviation for the entire sample period, we use 12 months standard deviation while estimating the EMP in our study. Therefore σ∆e and σ∆r will change after 12 months.

### Choice of variables in the estimation model and data sources

The choice of variables is well documented in the literature. US inflation rate (or US monetary policy) is one of the powerful determinants of global spillover (Georgiadis 2016). Both variables are included in our estimation as global factors. The regional influence can be captured by exchange rate changes of Russia, China and the Euro region. As explained in the previous section, the EMP method has been used to study regional exchange market pressure on CAEs. The changes in broad money (M3) provide a piece of important information on domestic inflation, credit spread and growth prospects (Berry et al. 2007). The inclusion of changes in broad money along with the changes in the trade balance is justified to control the exchange market pressure due to domestic macroeconomic fundamentals.

The data is obtained from the International Financial Statistics (IFS) of International Monetary Fund (IMF). The data covered the period from 2000Q1 to 2020Q4 related to four countries from central Asia i.e., Kazakhstan, Kyrgyzstan, Tajikistan and Uzbekistan. We gathered information related to the current account, foreign exchange reserves (excluding gold), exports, imports, broad money stock (M2), the exchange rates of four Central Asian countries, Russia and China. All these currencies represented monetary values against the dollar. Table 1 defines our variables, sources and summary statistics.

**Table 1:** Variable definitions and summary statistics

Variables	Definition	Obs	$\overline{X}$	σ
EMP Central Asia	Exchange market pressure index	280	1.778	5.738
EMP China	Exchange market pressure index of China	332	1.182	2.564
EMP Russia	Exchange market pressure index of Russia	332	1.051	1.545
Dollar Index	Dollar index against basket of currencies EUR, JPY,GBP, CAD, CHF, SEK	336	90.680	11.373
Exchange rate	Exchange rate of CA against dollar	336	760.38	1854
Current account	In million dollars	261	− 152.5	1053.3
Reserves	Official reserve assets in million dollars	282	9646	12,287
Broad money	In million dollars (M2)-logged	241	12.35	3.737
Exports	In million dollars	261	4073	6351
Imports	In million dollars	261	2999	3512

### Theoretical description of the empirical model

Several studies ((see Gevorkyan 2019; Gachunga 2019; Aizenman and Binici 2016) used system GMM estimation for studies related to finance. In order to estimate the effect of the interdependency of exchange rates of central Asian economies, Russia and China on the exchange market pressure index, we deployed two empirical strategies. First, we used system GMM analysis using unbalance panel data from 2000Q1 to 2020Q4 related to four central Asian economies. We deployed system GMM estimation method (Arellano and Bover 1995; Blundell and Bond 1998). Using this estimation method, the cross-section dimension of dataset used to minimize Hurwicz bias on lagged dependent variable. System GMM is panel estimation procedure assuming unit fixed effects (in our case country fixed effects). This empirical approach allow us to treat the endogeneity bias which matters for some our variables (see Roodman 2009; Soto 2009). In our estimation (see Model 1), we consider exchange market pressure, exchange rate, exports and imports as endogenous (their instruments include one lag period). Meanwhile, exchange market pressures for China, Russia, dollar index and exchange rate of CA economies against are considered weak endogenous (excluding lags). For specification check, the test of serial correlation (Arellano-Bond test) failed to reject the null hypotheses and we conclude that our specification does not suffer from serial correlation problem. To check instruments validity, we use Sargan J test which shows that our over-identifying restrictions are valid (see Chi2 test value with p-values in Table 3). In other words, we have more instruments than endogenous variables.^4^ We used heteroskedasticity-robust standard errors in our estimation.

Our baseline regression equation is;

5 $$\begin{aligned} EMP_{ijt} = & \emptyset EMP_{t - 1} + \beta_{1} EMP\_China_{ijt} \\ & + \beta_{2} EMP\_Russia_{ijt} + \beta_{3} Dollar\_Index_{ijt} \\ & + \beta_{4} ER\_CA_{ijt - 1} + \beta_{5} X_{ijt} + D_{i} + D_{t} + u_{ijt} \\ \end{aligned}$$

Equation 5 present the model of exchange market pressure across central Asian economies. EMP shows exchange market pressure with subscripts (i,j.t) for observation, country and in time t. EMP_China shows the exchange market pressure of China, while EMP_Russia provide the exchange market pressure for Russia. The dollar index represents the basket of currencies of the dollar against JPY, Euro, GBP etc. while ER_CA lagged in period^5^ show the exchange rates of central Asian economies against the dollar. The variable X represents the control variables such as reserves, broad money (M2), exports and imports. In order to capture the country and time fixed effects in our empirical analysis we used the set of country and year dummies (D).

## Results and discussions

### Exchange market pressure indexes for CAEs–results of estimated indexes

**Fig. 3:**
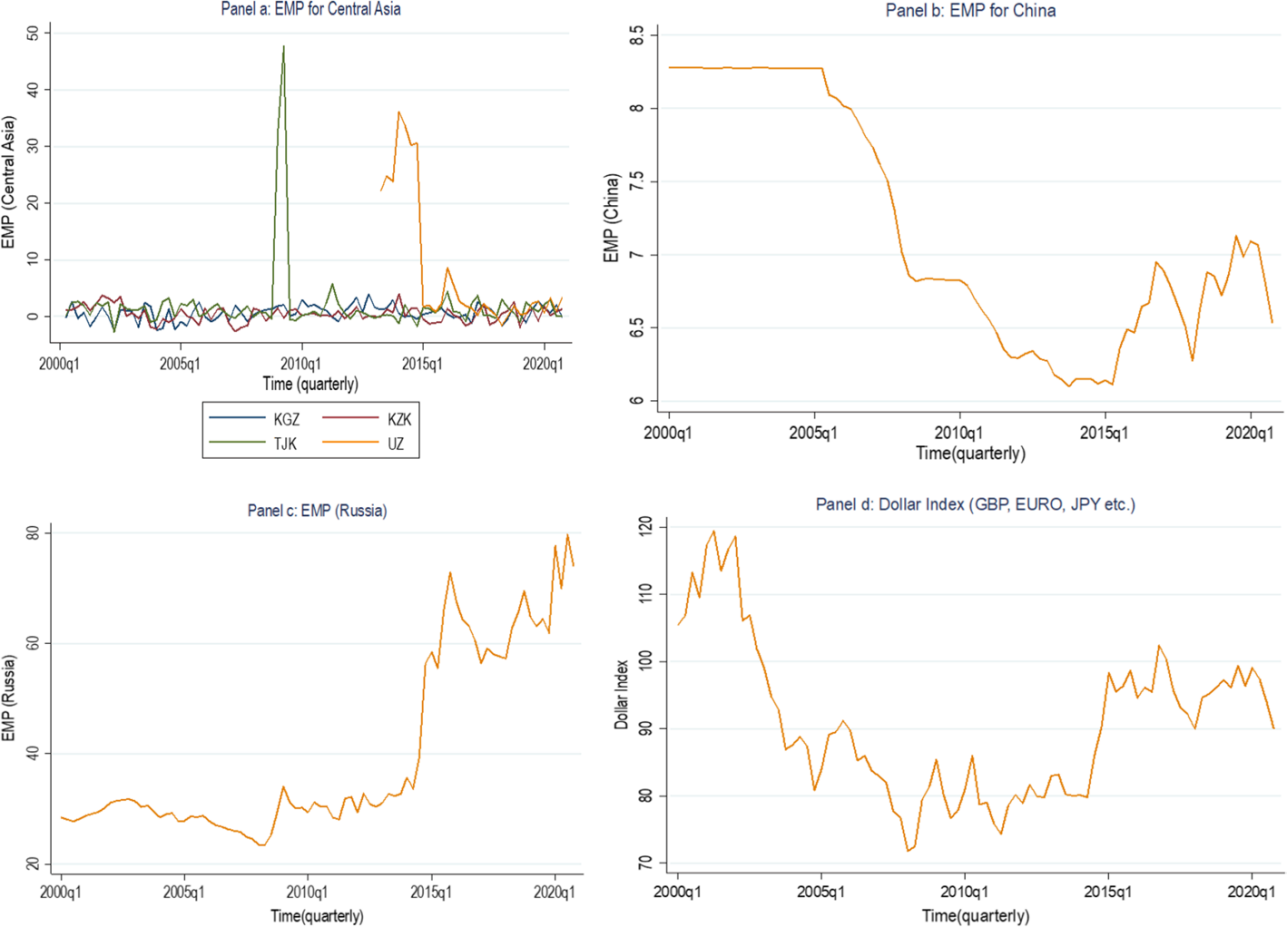
Exchange market pressures for CAE, China and Russia

In Fig. 3 we can see that the exchange market pressure index across CAEs show relatively low volatility compared to China and Russia. In the second panel 2, the graph shows that China EMP has high-level volatility between 2002q1 and 2005q1 and then it eases onwards. In comparison, Russia EMP volatility is more stable than China. On the other hand, the dollar index reduced significantly against other leading currencies such as the British pound, Euro, JPY etc. Overall, these graphs imply that China and Russia accumulate larger reserves by selling domestic currency to buy foreign exchange (US$) which result in high volatility for the Chinese and Russian exchange market pressures. While the dollar index reduced compared to other global currencies such as the British pound, Euro, JPY etc.

**Fig. 4:**
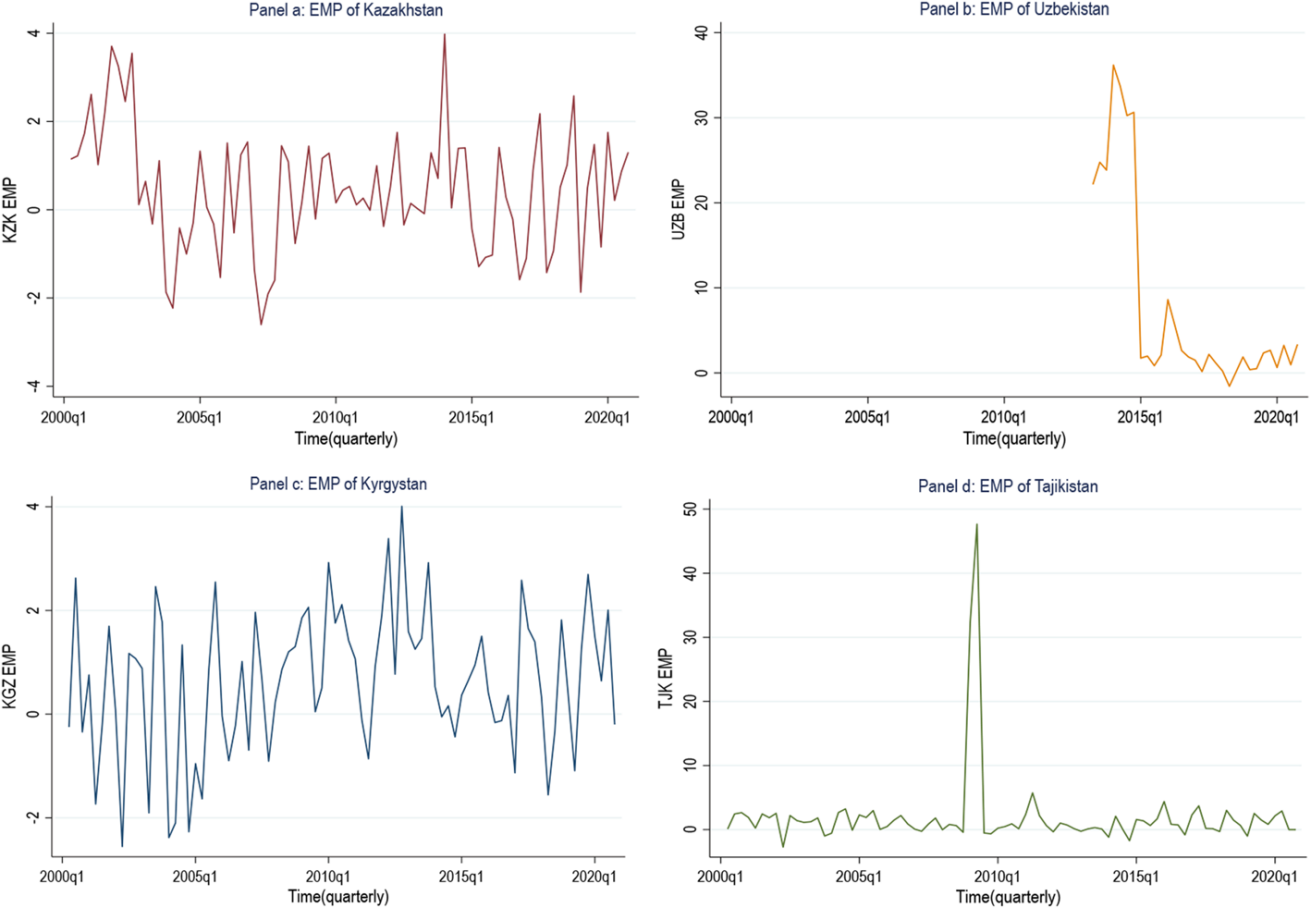
The exchange market pressure index of four CAEs

Based on these indexes (Fig. 4), we can get first impression of the foreign exchange pressures in these countries that includes exchange market volatility, balance of payment crises and other financial distresses. The crisis index (binary form) recommended by Eichengreen and Rose (1999) is widely used in the literature as crisis index indicating macrofinancial distress in the foreign exchange market.. The binary form of the index of Eichengreen and Rose (1999) as follows:

$${\text{Macro}} - {\text{financial}}\;{\text{distress}}/{\text{Crisis}}\;{\text{Index}} = {1}\;{\text{if}}\;{\text{EMPI}}_{{\text{t}}} > {\text{mean}}\;{\text{EMPI}}_{{\text{t}}} + {1}.{5}\; \sigma {\text{EMPI}}_{{\text{t}}} ,\;{\text{or}}\;{\text{else}} = 0$$

Based on this binary index, we can identify the periods of financial crisis from the above methodology. Econometrically, these EMPs of CAE are dependent variables for the estimation in the following section.

### Unit root test

In order to check whether our variables are stationary, we used Im et al. (2003) test (henceforward, IPS). This test works well for unbalance^6^ macro and micro-econometric panel datasets and assumes that the error terms are independently distributed normal for all *i* and *t*. Under null hypothesis (${H}_{0}:\varnothing i=0)$ of this test, we assume that panels contain a unit root, while rejecting the null hypothesis means that all panels follows the stationary process. For example, see below the definition of IPS (2003) test as follows

$$\begin{aligned} & H_{0} :all\;panels\;contain\;unit\;root \\ & H_{a} :At\;least\;one\;panel\;is\;stationary \\ \end{aligned}$$

In Table 2, we reported the Z-t-tilde-bar test values for all variables, this test value has an asymptotic standard normal distribution which is the standardized version of this statistics. From Table 2, we strongly reject the null hypothesis that all series contain a unit root. In other words, our data variables are stationary and failed to provide the evidence of spurious relationship.

**Table 2:** Unit root test (IPS, 2003)

Variables	$Z_{{\tilde{t} - bar}}$
EMP Central Asia	− 7.4008***
EMP China	− 6.0734***
EMP Russia	− 8.7952***
Dollar index	− 12.0571***
Exchange rate	− 11.2998***
Current	− 6.3822***
Reserves	− 10.3811***
Broad money	− 1.9521**
Exports	− 3.7736***
Imports	− 2.8009***

**P < 0.05; ***P < 0.01

### Discussion of results of system GMM

Table 3 provide information related to the impact of exchange market pressure across central Asian economies. The lagged value of the dependent variable is used as an instrument to address the endogeneity issue in our econometric model. This outcome indicates that past high-level pressure of CAE exchange rate would accelerate the volatility in the current exchange market pressure. The parameter of the exchange market pressure of China negatively affects the exchange market pressure of central Asian economies (CAEs). This is probably due to the link between trade and changes in the exchange rate of China (Garcia-Herrero and Koivu 2008). Since China is unsurprisingly running a huge trade surplus with CAEs (Contessi 2016), appreciating Chinese currency decreases the value of trade with CAEs thereby easing exchange market pressure in CAEs. Garcia-Herrero and Koivu (2008), have estimated that China’s import component (that is used as input for the exports to the western world) from East Asian countries will fall by 6% if the Chinese r*enminbi* appreciates by 10%.. Our finding has confirmed the similar impact. This outcome is robust across both models (see baseline model and model-1). Similarly, the Russian exchange market pressure also negatively influence the exchange market pressure across CAEs due to the huge trade surplus of Russia against CAEs. By looking into Fig. 2, CAEs has relatively developed a large trade deficit against Russia due to former supply chain links and Soviet-era trade structure. The influence of rubble and the US dollar on Central Asian Currencies have been well documented in the literature (for example, Shamshiev 2015). In comparison, the parameter of the dollar index shows a positive effect on the exchange market pressure of CAEs. This outcome suggests that if the dollar appreciates against other leading currencies such as Euro, GBP, JPY, CAD etc. it would increase the exchange market pressure across central Asian countries.The reserve composition has close link with currency movements and trade invoicing (Ito,McCauley and Chan, 2015). The elasticities of broad money without lag shows a positive influence on exchange market pressure across CEAs. This outcome indicates that a high volume of the money supply would increase exchange market pressure across CAEs. In other words, an increase in broad money through credit expansion in the domestic market would not only accelerate growth but also increase inflation which would result in high exchange market pressure across CAEs.. However, using the lagged broad money (− 1) present a negative effect on exchange market pressure. This finding shows that a high volume of broad money takes time (at least for one period) to ease the exchange market pressure across CAEs.

**Table 3:** System GMM analysis of EMP across central Asia

Variables	Baseline Model		Model-1	
EMP central Asia (depend.)	Coefficient (s.e)	t-value	Coefficients (s.e)	t-value
EMP (t − 1)	0.5526** (0.1268)	4.3	0.5183*** (0.1017)	5.0
EMP China	− 0.6415** (0.1478)	− 4.3	− 0.4977** (0.1353)	− 3.6
EMP Russia	− 1.2821** (0.5618)	− 2.2	− 1.0350** (0.3123)	− 3.3
Dollar Index	0.1601** (0.0594)	2.6	0.1195** (0.0332)	3.5
Exchange rate (t − 1)	− 0.0006** (0.0002)	− 3.0	− 0.0005** (0.0001)	− 5.0
Current	− 0.000 (0.000)	− 0.0	0.0002 (0.0003)	0.6
Current (t − 1)	–	–	− 0.0003 (0.0012)	− 0.2
Reserves	0.0000 (0.0000)	− 0.0	0.0001** (0.0000)	3.3
Reserves (t − 1)	–	–	− 0.0002* (0.0000)	− 2.2
Broad money	− 1.2801 (0.7165)	− 1.7	7.5201** (2.4296)	3.0
Broad money (t − 1)	–	–	− 8.1396** (2.5025)	− 3.2
Exports	0.0000 (0.0000)	0.0	0.0000 (0.0000)	0.0
Exports (t − 1)	–	–	0.0000 (0.0000)	0.0
Imports	0.0001 (0.0006)	0.1	− 0.0000 (0.0000)	− 0.0
Imports (t − 1)	–	–	0.0001 (0.0014)	0.0
Time dummies	[YES]	–	[YES]	–
Country dummies	[YES]	–	[YES]	–
AR (1) test [*P*-value]	[0.073]	–	[0.057]	–
AR (2) test [*P*-value]	[0.194]	–	[0.208]	–
Sargan test value [*P*-value]	Chi (153) = 174.99 [0.1079]	–	Chi (137) = 139.05 [0.4349]	–
Observations	234	–	223	–
Countries	4	–	4	–

*/**/***Significant at 1%; 5%; 10%. Robust standard errors are in parentheses

## Concluding remarks and policy implications

The role of managed float as a macroeconomic development strategy has been dealt with in the literature extensively. The latest research on regime role (Bleaney et al. 2018) suggests that less flexible exchange rate regimes or hard pegged arrangements have more negative impact on the growth and development compared to managed float. Hlédik et al. (2018) have analyzed the impact of fixed exchange rate regime on Kazakhstan’s macroeconomic development and monetary policy transmission. There are ample research studies on the trade surplus economies gradually moving from fixed exchange rate regime to managed float. The Kazakhstan has moved to managed float in 2015. There are other dynamics for exchange rate regime changes as well. Not all trade surplus economies will benefit from the change from fixed to managed float. Towbin and Weber (2013) suggest that if a country is having high foreign debt, it will erode the benefits of flexible exchange rate regimes.

Flexible exchange rate policy does not necessarily translate into the independence of monetary policy or monetary policy autonomy (Rey 2018). Financial shocks in form of exchange rate pass-through have impacted transition economies and emerging economies irrespective of exchange rate regime, state of economy and trade links (Corsetti et al. 2021). The analysis of exchange market pressure in CAEs assumes importance as CAEs have started towards inflation targeting regime. It adds to the existing monetary policy challenges. We argue that before devising an appropriate exchange rate policy, there should be an analysis of exchange market pressure emanating from global sources and regional sources through trade and other cross border transactions.

For this analysis, we created an exchange market pressure index for each CAE. We also created an exchange market pressure index for the Rubble and Renminbi (to capture regional interdependence of foreign exchange market).

The estimation of the Exchange Market Pressure Index for four Central Asian economies is a unique contribution to the literature. For the first time in the literature, the issue of interdependence in the CAE foreign exchange market with major trade blocs has been addressed in depth. For an intuitive grasp of the matter, we have also explored the expanding pattern of trade among CAEs and with the four largest trading partners. As mentioned earlier, the data availability for CAEs is not robust and that is the limitation of the study.In view of current geopolitical situation (Russia-Ukraine war), there is a further scope to study the impact of currency movements from major trading partners. It will assist policy makers of CAEs to realign their exchange rate mechanism in order to ward off excessive negative impact of financial market turmoils of recent time.

We analyzed past monetary policy conducts of CAEs. Our findings suggest a framework to institute an appropriate exchange rate policy framework in CAEs. It contributes to the growing discussion on monetary policy challenges in transition economies of Central Asia.

## Data Availability

The data set use and analyzed during the current study are available from corresponding author on reasonable request.

## Appendix

See Table 4.

**Table 4:** Sketch view of macroeconomic profile of CAEs (As on Dec 2020). *Source*: Asian Development Bank, Key Indicators for Asia and Pacific 2021 accessed on 28th June 2022)

SN	Macro indicator	Kazak	Tajakistan	Krygk	Uzbek	Turkinistan
1	GDP	(−) 2.6%	7.40% (2019)	(−) 8.6%	1.6%	6.30% (2019)
2	Inflation/CPI	6.8%	7.40% (2019)	6.3%	12.9%	10%
3	Exchange Rate (average period)	413	10.32	77.35	10,094.2	3.5
4	Interest rate (6 to 12 months)	7.4%	11.5% (2019)	6.4%	17.2%	NA
5	Unemployment rate	4.9%	2.1% (2019)	5.8%	0.30%	4%
6	External Debt to GNI	98.30	70.20	106.40	37	2.3% (2018)
7	Trade balance/GDP	6.1%	(−) 18.10%	(−) 18.4%	(−) 10.8%	5% (2019)
8	Current A balance/GDP	(−) 3.7%	4.20%	(−) 4.5%	(−) 5.4%	1.3% (2019)

## Abbreviations

CBU: Central bank of Uzbekistan
CAEs: Central Asian economies
DCP: Dominant currency Paradigm
EMP: Exchange market pressure
EMPI: Exchange market pressure index
GMM: Generalized method of moments
IMF: International monetary fund
IFS: International financial statistics
I P S: Im, Pesaran and Shin
OLS: Ordinary lease square

## Footnotes

1 Consists of five countries- Kazakhstan, Kyrgyzstan, Tajikistan, Turkmenistan and Uzbekistan.

2 Turkmenistan is excluded due to non-availability of time-series data. Other researcher (Poghosyan 2021) has also faced the same problem of getting sufficient data on Turkmenistan.

3 the detailed discussion can be found at https://raexpert.ru/researches/int_fin/central_asia_2021/ “The macroeconomic situation in countries of Central Asia”.

4 Using the difference of exchange market pressure variables, broad money stock, current account, export and imports etc. as instruments provide efficient estimates in dynamic models for panel datasets (for detail see, Wooldridge 1996).

5 In order to examine the impact of exchange rates of CAEs (against dollar) on exchange market (EMP), we included the lag for one period which is customary in macroeconomic literature (see Pissarides and Vallanti 2007) to avoid the endogeneity issue and second, the domestic exchange rate (against) take at least one year time to have an effect on their EMP.

6 Our panel data is unbalanced, and we are left with this choice to use IPS (2003) test.
